# Prevalence Rates and Predictors of Generalized Anxiety Disorder Symptoms in Residents of Fort McMurray Six Months After a Wildfire

**DOI:** 10.3389/fpsyt.2018.00345

**Published:** 2018-07-31

**Authors:** Vincent I. O. Agyapong, Marianne Hrabok, Michal Juhas, Joy Omeje, Edward Denga, Bernard Nwaka, Idowu Akinjise, Sandra E. Corbett, Shahram Moosavi, Matthew Brown, Pierre Chue, Andrew J. Greenshaw, Xin-Min Li

**Affiliations:** ^1^Department of Psychiatry, Faculty of Medicine, University of Alberta, Edmonton, AB, Canada; ^2^Addiction and Mental Health, Alberta Health Services, Edmonton, AB, Canada; ^3^Department of Public Health, Alberta Health Services, Fort McMurray, AB, Canada; ^4^Department of Family Medicine, Faculty of Medicine, University of Alberta, Edmonton, AB, Canada; ^5^Department of Psychiatry, Northern Lights Regional Health Centre, Fort McMurray, AB, Canada

**Keywords:** wildfire, major depressive disorder, mental health, anxiety disorder, support, counseling

## Abstract

The Fort McMurray wildfire was the costliest disaster in Canadian history, with far-reaching impacts. The purpose of this paper is to examine the prevalence and risk factors of elevated generalized anxiety disorder (GAD) symptomatology in residents of Fort McMurray 6 months after the wildfire. Data were collected via random selection procedures from 486 participants. Generalized anxiety disorder symptoms were measured via the GAD-7. The 1-month prevalence rate for GAD symptomatology 6 months after the disaster was 19.8% overall, regression analyses revealed six variables with significant unique contributions to prediction of GAD symptomatology. Significant predictors were: pre-existing anxiety disorder, witnessing of homes being destroyed by the wildfire, living in a different home after the wildfire, receiving limited governmental support or limited family support, and receiving counseling after the wildfire. Participants with these risk factors were between two to nearly seven times more likely to present with GAD symptomatology. In addition, participants who presented with elevated symptomatology were more likely to increase use or problematically use substances post-disaster. This study extends the literature on mental health conditions and risk factors following disasters, specifically in the area of generalized anxiety. Findings and implications are discussed.

## Introduction

The 2016 Fort McMurray wildfire was the costliest natural disaster in Canadian history^1^. The wildfire had far-reaching impacts. It destroyed hundreds of homes and businesses, hundreds of thousands of acres of land, and necessitated the largest evacuation in Alberta's history, relocating thousands of residents of Northern Alberta. The objective of this study was to examine the prevalence of generalized anxiety disorder (GAD) symptoms and associations with sociodemographic, exposure-related, and clinical risk factors in survivors of the fire.

Over 40% of Canadians will be exposed to a major disaster in their lifetime, with the majority reporting significant impact on their ability to function in day-to-day life (1). Although exposure to natural disasters is relatively common, a subset of persons exposed will struggle with clinically significant mental health conditions, such as post-traumatic stress disorder (PTSD), depression, and substance use disorders (2). Research suggests risk factors that may place people experiencing disasters at greater risk of incurring mental health conditions (3). For example, in a survey of veterans who survived Hurricane Katrina in the Gulf Coast of the United States, persons with a pre-existing mental condition, especially PTSD, were nearly 12 times more likely to develop another mental health condition on screening following the disaster (4). However, the relative risk of identified sociodemographic and clinical factors in predicting post-disaster mental health symptomatology such as PTSD symptoms is unclear, with some inconsistencies in results reported which may be related to methodological factors (5).

Most post-disaster research has focused on PTSD and depression, with little research examining prevalence and risk factors associated with the development of post-disaster anxiety symptoms (2). Published research suggests that GAD symptoms are elevated following disasters (6–9). In addition, prevalence rates of GAD tend to remain stable over time rather than showing attenuation, as is often reported in PTSD research (6, 9, 10). Of the existing research, GAD symptoms have been associated with pre-existing mental health conditions as well as exposure to disaster-related variables, such as high typhoon exposure (11, 12). Some variables, such as degree of exposure to the disaster, may confer increased risk for the development of anxiety symptoms, whereas other variables, such as higher income, may exert a protective effect (13). Gene by environment interactions have also been reported, such that specific genetic factors differentially increase the risk of the development of GAD in individuals following disasters (8, 14). Conceptually therefore, genetic, social, environmental, and psychological factors could combine to explain elevated levels of GAD following wildfires. “Feelings of uncertainty” which is a central feature of the disaster experience (15) could be lead to increased anxiety levels post-disasters. Uncertainty around housing, job security, and the stress of dealing with insurance companies as well as concerns related to the long-term damaging physical health effects of the smoke from the wildfires could all culminate in elevated anxiety levels post-disaster. Furthermore, the possibility of recurring wildfires could heighten the uncertainty and increase anxiety levels in the victims. Other studies have suggested increased use of nicotine, alcohol and other substances following natural disasters and that the co-existence of substance use with mental health disorders worsens the outcome for survivors (16–19).

To add to the limited literature related to post-disaster anxiety disorders, our study aims to examine the impact of the 2016 Fort McMurray wildfires on the prevalence of elevated GAD symptoms and examine whether sociodemographic, exposure-related, and clinical factors are associated with elevated symptomatology. Consistent with previous literature regarding mental health conditions following a disaster, we hypothesize that there will be increased prevalence rates of GAD symptomatology in the respondents compared to what they self-report prior to the wildfires. In addition, we hypothesize that sociodemographic, exposure-related, and clinical variables would predict the likely presence of GAD symptoms. Another goal of this study is to investigate the association between likely GAD and substance use. We hypothesize that consistent with the literature on the mental health and substance use effects on victims' post-disasters, we will observe increased substance use in respondents who had likely GAD.

## Materials and methods

Quantitative data were collected using self-administered paper-based questionnaires. Study participants were selected using random selection procedures. Participants completed written informed consent before completing the survey questionnaires. This study was carried out in accordance with the recommendations of the University of Alberta Review and Ethics Board. The protocol was approved by the University of Alberta Review and Ethics Board (Pro00066054). All subjects gave written informed consent in accordance with the Declaration of Helsinki.

A targeted sample size of 1,050 was calculated based on an adult population estimates of 60,000 for Fort McMurrary 6 months after the fire, a 95% confidence interval, and a margin of error of ±3% for estimates of the prevalence rates for GAD. Based on this sample size estimate and an assumption of a dropout rate approximating 30%, random selection procedures were used to distribute survey questionnaires to 1,500 adult residents of Fort McMurray. Data were collected in November 2016. Data collection sites were located at various sites within the community and included: a main recreation center, a post-secondary educational institution, a public library, and from three large religious congregations. In each facility, adult residents were randomly approached and invited to participate in the study. Prospective respondents were offered the opportunity to complete the survey forms at the designated data collection points or to take them home and return completed forms within a week to a data collection point. Overall, 1,500 survey forms were randomly distributed during the data collection period. All adult residents who were approached indicated their acceptance of the invitation to participate by collecting the survey forms although not all of them completed and/or returned the completed forms.

A data collection form was designed and used to collect predictive sociodemographic and clinical information, exposure-related data, and support information from respondents. Selected predictive factors were based on a literature review of the factors which have been previously investigated in relations to the mental health effects (depression, anxiety and PTSD) of wildfires and other natural disasters (16, 17, 20–25). The GAD-7 was used to assess GAD symptomatology (26). According to these authors, sensitivity and specificity of the GAD-7 exceed 0.80 at a cut point of 10 or greater “at which threshold sensitivity is nearly maximized”. The cut-off point we used for probable presence of GAD in this study was 10 on the GAD-7.

Substance use was assessed using the Alcohol Use Disorder Identification Test [AUDIT; (27)], the Drug Use Disorder Identification Test [DUDIT; (28)] and the Fagerstrom Test for Nicotine Dependence (29).

SPSS Version 20 (30) was used to analyze data. Univariate analyses with Chi-squares tests were used to ascertain the relationship between predictor variables and GAD symptomatology. Predictor variables that showed statistically significant relationships (*p* ≤ 0.05, two-tailed) with GAD on univariate analysis and predictor variables that trended toward significance (0.05 ≤ *p* ≤ 0.1, two-tailed) were then entered into a logistic regression model. Prior to performing the logistic regression analysis, correlational diagnostics were performed to identify any strong inter-correlations (Spearman's correlation coefficient of 0.7–1.0 or −0.7–−1.0) among predictor variables. Consequently, “*sought counseling after the wildfire' which was highly positively correlated with “received counseling after the wildfire,” (Spearman's correlation coefficient of 0.80*) was dropped from the regression model. Furthermore, “*on no psychotropic medication”* was also dropped from the regression model as it was highly negatively correlated with “*on antidepressants before the wildfire”* (Spearman's correlation coefficient of −0.80). Using this approach, we avoided multicollinearity. All other variables in the model were not as strongly correlated with each other. Odds ratios from the binary logistic regression analysis were calculated to determine the association between the predictor variables and GAD symptoms, controlling for the other variables in the model. Finally, we used Chi-square tests to ascertain the relationship between GAD symptomatology and substance use. In other to determine the unique contribution of GAD symptomatology to substance use in the respondents independent of their history of a mental health diagnosis and usage of psychotropic medication before the wildfire” we used a logistic regression model to examine the impact of GAD on substance use whilst specifically controlling for pre-existing mental health conditions and use of psychotropic medication before the wildfires.

## Results

Results will be presented as follows. First, descriptive characteristics of the sample (e.g., sociodemographic, living situation, exposure to disaster, clinical variables) will be presented. Second, the associations between GAD symptoms and the descriptor variables will be discussed. Third, the results of logistic regression examining significant predictors of GAD symptomatology will be presented, as well as the influence of GAD symptomatology on self-reported substance use.

### Descriptive sample characteristics

Sample characteristics are as listed in Table 1. The sample was predominantly 26 years of age or older (75%), female (67%), employed or in school (87%), and married, partnered, or cohabiting (64%). Most participants were living in the same home they lived in before the fire (79%). Although many reported no significant loss of property or business due to the wildfire (48%), the majority of participants reported a high degree of exposure to the wildfire. For example, most were in town during the evacuation (92%), witnessed houses burning (69%), and were in close proximity to property destruction (70%). The majority of participants reported daily exposure to media coverage of the wildfire and its effects (i.e., 80, 88%) and 81% feared for their lives or their family members' lives.

**Table 1:** Descriptive Characteristics^*^ of the Sample.

**Variable type**	**Variables**	**Overall**
Sociodemographic	**Age (Years)** < 25 26–40 >40	119 (24.6%) 189 (39.0%) 176 (36.4%)
	**Gender** Male Female	162 (33.4%) 322 (66.5%)
	**Employment status** Employed Unemployed Student	313 (64.7%) 66 (13.6%) 105 (21.7%)
	**Relationship status** Married/cohabiting/partnered Single/separated/divorced/widowed	302 (63.7%) 172 (36.3%)
Living situation	**Where respondents live after the fire** Own home Renting or other accommodation	282 (58.4%) 201 (41.6%)
	**Where respondents lived after the wildfire relative to where they lived before the wildfire** Same home they lived in before the fire Different home although previous home was not destroyed by the fire Different home because previous home was destroyed by the fire	383 (79.3%) 51 (10.6%) 49 (10.1%)
Exposure to disaster	**Where respondent was on the day of the evacuation** At home At work/school/shops or running errands in town Out of town	222 (45.9%) 224 (46.3%) 38 (7.9%)
	**Area of residence relative to destroyed properties** 0–1.0 properties destroyed per kilometer square 1.1–50.0 properties destroyed per kilometer square 50.1–300.0 properties destroyed per kilometer square	107 (22.2%) 336 (69.6%) 40 (8.3%)
	**Respondents witnessed burning of homes by the wildfires**	331 (68.8%)
	**Respondents were fearful for their lives or the lives of friends/family**	388 (81.2%)
	**How frequently did respondents watch television images about the devastation caused by the wildfires during the period of the evacuation** Daily Less frequently than daily	424 (87.8%) 59 (12.2%)
	**How frequently did respondents read newspaper and internet articles related to the devastation caused by the wildfires** Daily Less frequently than daily	390 (80.4%) 95 (19.6%)
	**Home was completely destroyed by the wildfire**	53 (11%)
	**Home suffered substantial smoke damage**	51 (10.6%)
	**Suffered no loss of property or business from the wildfire**	234 (48.4%)
Clinical history	**Respondent had a history of depressive disorder before the wildfire**	52 (10.8%)
	**Respondent had a history of anxiety disorder before the wildfire**	69 (14.4%)
	**Respondent had no history of mental health diagnosis before the wildfire**	383 (79.8%)
	**Respondents were on antidepressants before the wildfire**	46 (9.6%)
	**Respondents were on sleeping medication before the wildfire**	19 (4.0%)
	**Respondents were on mood stabilizers before the wildfire**	14 (2.9%)
	**Respondents were on no psychotropic medication before the wildfire**	412 (85.8%)
Support	**Received sufficient support from family and friends** Yes, absolute support Yes, some support Yes, but only limited support Not at all	318 (65.8%) 112 (23.2%) 38 (7.9%) 15 (3.1%)
	**Received sufficient support from the Red Cross** Yes, absolute support Yes, some support Yes, but only limited support Not at all	219 (45.5%) 183 (38%) 68 (14.1%) 11 (2.3%)
	**Received sufficient support from the government** Yes, absolute support Yes, some support Yes, but only limited support Not at all	163 (34.2%) 170 (35.7%) 88 (18.5%) 55 (11.6%)
	**Received sufficient support from insurers** Yes, absolute support Yes, some support Yes, but only limited support Not at all Not Applicable as respondent did not have insurance	191 (40.7%) 123 (26.2%) 50 (10.7%) 34 (7.2%) 71 (15.1%)
Post-crisis counseling	**Sought counseling after the wildfire**	64 (13.3%)
	**Received counseling after the wildfire**	67 (13.9%)
Objective measures	**Respondents had elevated symptoms consistent with GAD** (based on GAD-7 scale)	96 (19.8%)
	**Alcohol Use Identification Test (AUDIT)** ≤ 7 (low risk drinking or abstinence) ≥8 (High risk, harmful or hazardous drinking or alcohol dependence)	416 (86%) 68 (14%)
	**Drug Use Identification Test (DUDIT)** ≤ 5 for men and ≤ 1 for women(No drug related problems) ≥6 for men and ≥2 for women (Drug related problems)	435 (89.7%) 50 (10.3%)
	**Fagerstrom Test for Nicotine Dependence** ≤ 4 (low to moderate dependence) ≥5 (moderate to high dependence)	453 (93.4%) 32 (6.6%)

* *Demographic and clinical characteristic were all self-reported*.

In terms of clinical history, most participants reported absence of a mental health condition prior to the wildfires (80%), with few on psychotropic medication prior to the wildfire (86%). On objective measures, one-fifth of the sample had elevated GAD symptoms (GAD-7, 20%), and a minority reported high levels of substance use (7–14%). A minority sought counseling after the fire (13%), with nearly all of those who sought counseling receiving it. Most participants reported receiving some or absolute support from a variety of sources, including family and friends (89%), the Red Cross (84%), the government (70%), and insurance companies (67%).

### Associations between sociodemographic, clinical, exposure-related, and support variables and elevated GAD symptoms

Chi-square analyses (see Table 2) suggested that persons with elevated GAD symptomatology were more likely to be young, female, staying in rental or other accommodation, and living in a home after the wildfire that was different than the home they were living in prior to the wildfire. Participants with elevated GAD symptoms were more likely to read wildfire-related media. In terms of clinical variables, participants with elevated GAD symptoms were more likely to have a history of a mental health condition or have been taking psychotropic medications prior to the wildfire. They were more likely to seek counseling, and less likely to report receiving adequate support, both informal and formal.

### Predictors of elevated GAD symptoms

The results of the Chi-square analysis were used to inform the selection of variables to be included as potential predictors in a logistic regression analysis. Specifically, nineteen of the variables identified via Chi-square analysis (see Table 2) with significant *p*-values (*p* ≤ 0.5) or *p*-values that were trending or approaching significance (*p*-values of 0.05–0.1), were entered into a logistic regression model. Note that the variable “sought counseling after the wildfire” was not included in the model, because it was highly correlated with the variable “received counseling after the wildfire” (Spearman correlation = 0.80). Given the high level of conceptual and statistical similarity, this variable was considered redundant and was eliminated.

**Table 2:** Chi-Square Analyses of Relationships between Variables^*^ and GAD Symptoms.

**Variable type**	**Variables**	**GAD likely**	**Chi-square value**	***P-*value**
Sociodemographic	**Age (Years)**			
	< 25 26–40 >40	35 (29.4%) 30 (15.9%) 31 (17.5%)	9.34	**0.01**
	**Gender**			
	Male Female	24 (14.7%) 72 (22.4%)	3.97	**0.05**
	**Employment status**			
	Employed Unemployed Student	49 (15.6%) 17 (25.8%) 30 (28.6)	10.04	**0.01**
	**Relationship status**			
	Married/cohabiting/partnered Single/separated/divorced/widowed	41 (23.8) 53 (17.5%)	2.78	0.12
Living situation	**Where respondents live after the fire**			
	Own home Renting or other accommodation	47 (16.6%) 47 (24.4%)	4.46	**0.04**
	**Where respondents lived after the wildfire relative to where they lived before the wildfire**			
	Same home they lived in before the fire Different home although previous home was not destroyed by the fire Different home because previous home was destroyed by the fire	62 (16.1%) 19 (37.3%) 13 (26.5%)	14.58	**0.00**
Exposure to disaster	**Where respondent was on the day of the evacuation**			
	At home At work/school/shops or running errands in town Out of town	49 (22.0%) 41 (18.3%) 6 (15.8%)	1.36	0.51
	**Area of residence relative to destroyed properties**			
	0–1.0 properties destroyed per kilometer square 1.1–50.0 properties destroyed per kilometer square 50.1–300.0 properties destroyed per kilometer square	24 (22.4%) 59(17.5%) 13 (32.5%)	5.64	0.06
	**Respondents witnessed burning of homes by the wildfires**			
	No Yes	74 (22.3%) 22 (14.7%)	3.76	0.06
	**Respondents were fearful for their lives or the lives of friends/family**			
	No Yes	11 (12.2%) 83 (21.3%)	3.85	0.06
	**How frequently did respondents watch television images about the devastation caused by the wildfires during the period of the evacuation**			
	Daily Less frequently than daily	87 (20.5%) 9 (15.3%)	0.89	0.39
	**Frequency with which respondents read newspaper and internet articles related to the devastation caused by the wildfires**			
	Daily Less frequently than daily	86 (22.0%) 10 (10.5%)	6.34	**0.01**
	**Home was completely destroyed by the wildfire**			
	No Yes	55 (22.1%) 40 (17.1%)	1.74	0.21
	**Home suffered substantial smoke damage**			
	No Yes	84 (19.4%) 11 (26.1%)	0.14	0.71
	**Suffered no loss of property or business from the wildfire**			
	No Yes	81 (18.8%) 14 (26.4%)	1.84	0.20
Clinical history	**Respondent had a history of depressive disorder before the wildfire**			
	No Yes	66 (15.4%) 28 (53.8%)	43.63	**0.00**
	**Respondent had a history of anxiety disorder before the wildfire**			
	No Yes	60 (14.6%) 34 (49.3%)	45.29	**0.00**
	**Respondent had no history of mental health diagnosis before the wildfire**			
	Yes No	53 (13.8%) 41 (42.3%)	39.91	**0.00**
	**Respondents were on antidepressants before the wildfire**			
	No Yes	74 (17.0%) 20 (43.5%)	18.53	**0.00**
	**Respondents were on sleeping medication before the wildfire**			
	No Yes	83 (18.0%) 11 (57.9%)	18.50	**0.00**
	**Respondents were on mood stabilizers before the wildfire**			
	No Yes	87 (18.6%) 7 (50.0%)	8.51	**0.01**
	**Respondents were on** a psychotropic medication **before the wildfire**			
	No Yes	37 (9.6%) 31 (33.0%)	34.17	**0.00**
Support	**Received sufficient support from family and friends**			
	Yes, absolute support Yes, some support Yes, but only limited support Not at all	45 (14.2%) 32 (33.7%) 12 (31.6%) 6 (40.0%)	18.84	**0.00**
	**Received sufficient support from the Red Cross**			
	Yes, absolute support Yes, some support Yes, but only limited support Not at all	32 (14.6%) (20.1%) 19 (27.9%) 5 (45.5%)	11.26	**0.01**
	**Received sufficient support from the government**			
	Yes, absolute support Yes, some support Yes, but only limited support Not at all	19 (11.7%) 31 (18.1%) 22 (25.0%) 20 (36.4%)	18.39	**0.00**
	**Received sufficient support from insurers**			
	Yes, absolute support Yes, some support Yes, but only limited support Not at all Not Applicable as respondent did not have insurance	30 (15.7%) 23 (18.5%) 13 (226.0%) 7 (20.6%) 17 (23.9%)	4.11	0.39
Post-crisis counseling	**Sought counseling after the wildfire**			
	No Yes	69 (16.5%) 27 (42.2%)	23.06	**0.00**
	**Received counseling after the wildfire**			
	No Yes	68 (16.4%) 27 (40.3%)	20.85	**0.00**

* *Variables were self-reported including histories of metal disorders. Bold values are results that are statistically significant, associated probability displayed (criterion for type 1 error = P < 0.05)*.

See Table 3 for regression results. The full model including all nineteen predictors was statistically significant, χ^2^ (29, *N* = 486) = 106.05, *p* < 0.00, which suggested that the model was able to distinguish between participants with elevated GAD symptoms vs. those who did not report elevated symptoms. The model as a whole explained ~21–33% (Cox and Snell *R*^2^, Nagelkerke *R*^2^, respectively) of the variance in GAD-7 symptomatology and correctly classified 84% of cases.

**Table 3:** Predictors of GAD Symptomatology.

**Predictor**	**B**	**SE**	**Wald**	**df**	***P*-value**	**Odds ratio**	**95% CI for odds ratio**	
							**Lower**	**Upper**
Age (Years)								
>25 26–40 < 40	−0.81 −0.07	0.42 0.45	5.16 3.67 0.02	2 1 1	0.08 0.06 0.89	0.47 0.94	0.20 0.39	1.02 2.27
Gender								
Male Female	0.23	0.33	0.51	1	0.48	1.26	0.66	2.41
Employment status								
Employed Unemployed Student	0.74 0.76	0.45 0.41	5.02 2.68 3.41	2 1 1	0.08 0.10 0.07	2.09 2.14	0.87 0.96	5.04 4.78
Area of residence relative to destroyed properties								
0–1.0 properties destroyed per kilometer square 1.1–50.0 properties destroyed per kilometer square 50.1–300.0 properties destroyed per kilometer square	0.21 0.02	0.36 0.65	0.40 0.34 0.00	2 1 1	0.82 0.55 0.97	1.24 1.02	0.62 0.29	2.48 3.62
Where respondents lived after the fire								
Own Home Renting or other accommodation	−0.30	0.35	0.73	1	0.39	0.74	0.37	1.47
Respondents witnessed burning of homes by the wildfires								
No Yes	0.80	0.36	4.95	1	**0.03**	**2.22**	1.10	4.49
Respondents were fearful for their lives or the lives of friends/family								
No Yes	0.61	0.44	1.92	1	0.17	1.84	0.78	4.37
Frequency with which respondents read newspaper and internet articles related to the devastation caused by the wildfires								
Less frequently than daily Daily	0.64	0.46	1.92	1	0.17	1.90	0.77	4.69
Where respondents lived after the wildfire relative to where they lived before the wildfire								
Same home they lived in before the fire Different home- previous home not destroyed by the fire Different home-previous home destroyed by the fire	1.34 0.44	0.44 0.58	9.52 9.49 0.58	2 1 1	0.01 **0.00** 0.45	**3.82** 1.55	1.63 0.50	8.95 4.81
Respondent had a history of depressive disorder before the wildfire								
No Yes	1.18	0.62	3.67	1	0.06	3.26	0.97	10.95
Respondent had a history of anxiety disorder before the wildfire								
No Yes	1.91	0.72	7.04	1	**0.01**	**6.76**	1.65	27.70
Respondent had a history of mental health diagnosis before the wildfire								
No Yes	1.03	0.82	1.59	1	0.21	2.81	0.56	14.01
Respondents were on antidepressants before the wildfire								
Yes No	0.27	0.57	0.23	1	0.63	1.31	0.43	3.98
Respondents were on sleeping medication before the wildfire								
No Yes	0.249	0.72	0.12	1	0.73	1.28	0.31	5.25
Respondents were on mood stabilizers before the wildfire								
No Yes	−0.51	0.97	0.27	1	.60	0.60	0.09	4.01
Received sufficient support from family and friends								
Yes, absolute support Yes, some support Yes, but only limited support Not at all	0.59 1.02 1.24	0.33 0.48 0.87	6.94 3.19 4.45 2.05	3 1 1 1	0.07 0.07 **0.04** 0.15	1.81 **2.76** 3.45	0.94 1.07 0.63	3.45 7.08 18.82
Received sufficient support from the Red Cross								
Yes, absolute support Yes, some support Yes, but only limited support Not at all	−0.40 −0.50 −0.53	0.38 0.54 1.04	2.80 1.11 1.72 0.26	3 1 1 1	0.42 0.29 0.10 0.61	0.67 0.41 0.59	0.32 0.14 0.08	1.41 1.18 4.54
Received sufficient support from the government								
Yes, absolute support Yes, some support Yes, but only limited support Not at all	0.58 1.46 1.29	0.43 0.54 0.53	9.341 1.83 7.29 6.07	3 1 1 1	**0.03** 0.18 **0.01** **0.01**	1.79 **4.31** **3.65**	0.77 1.49 1.30	4.14 12.44 10.22
Received counseling after the wildfire								
No Yes	1.32	0.40	10.84	1	**0.00**	**3.74**	1.71	8.20
Constant	−5.78	1.18	24.13	1	0.00	0.00		

*Bold values are results that are statistically significant, associated probability displayed (criterion for type 1 error = P < 0.05)*.

However, only six of 19 predictors made unique contributions (i.e., witnessing of homes burning, place of residence after the wildfire, pre-existing anxiety disorder, perceived support from the government or family/friends, post-crisis counseling). Odds ratios varied from 2.22 (witness of homes burning) to 6.76 (pre-existing anxiety disorder). Participants who witnessed homes burning were approximately twice as likely to have elevated GAD symptoms on self-report, whereas persons who were relocated were nearly four times as likely to present with GAD symptoms. Perceived lower levels of support was also a predictor of GAD symptoms, specifically that persons receiving limited informal support (family and friends) were nearly three times as likely to report elevated GAD symptoms and those receiving limited governmental support were approximately four times more likely to report elevated anxiety symptoms. Participants who received post-crisis counseling were four times more likely to report elevated anxiety symptoms. Participants with a pre-existing anxiety disorder were nearly seven times more likely to report elevated GAD symptoms post-wildfire.

Not only was a pre-existing anxiety disorder a risk factor for elevated GAD symptoms, but participants with elevated anxiety symptoms post-wildfire were also significantly more likely to report increased or problematic levels of substance use (i.e., alcohol, drug, and nicotine, see Table 4).

**Table 4:** Impact of Elevated GAD Symptoms on Substance Use.

**Variables**	**GAD unlikely**	**GAD likely**	**Chi-square value**	***P*-value**
*Self-reported increased alcohol use	18 (31.0%)	40 (69.0%)	5.20	0.03
*Self-reported increased drug use	5 (35.7%)	9 (64.3%)	17.86	0.00
**Alcohol Use Identification Test Scores (AUDIT)**				
≥8 (High risk, harmful or hazardous drinking or alcohol dependence)	21 (30.9%)	47 (69.1%)	6.13	0.02
**Drug Use Identification Test Scores (DUDIT)**				
≥6 for men and ≥2 for women (Drug related problems)	22 (44.0%)	28 (56.0%)	20.67	0.00
**Fagerstrom Test for Nicotine Dependence**				
≥5 (moderate to high dependence)	12 (37.5%)	20 (62.5%)	6.81	0.02

* *Self-reports of increased use of alcohol and drugs were ascertained by asking respondents if their alcohol or drug use had increased after the wildfires compared to before the wildfires. The optional responses provided to this question was “Yes” and “No”*.

Table 4 suggests there was statistically significant elevation in the levels of alcohol, nicotine and substance use as measured by self-reports and standardized rating scales in respondents who had likely GAD compared to respondents who did not have GAD.

In order to determine the unique contribution of likely GAD to substance use in the population 6 months after the wildfire independent of a prior mental health diagnosis or use of psychotropic medication before the wildfire, we entered the three factors into a logistic regression model. The full model including all three predictors was statistically significant, χ^2^ (3, *N* = 486) = 25.35, *p* < 0.00, which suggested that the model was able to distinguish between participants with drug related problems vs. those who did not have drug related problems. The model as a whole explained only 5.2–10.8% (Cox and Snell *R*^2^, Nagelkerke *R*^2^, respectively) of the variance in drug related problems and correctly classified 89.9% of cases.

Table 5 suggests that likely GAD made a unique statistically significant contribution to the model. With an odds ratio of 2.88, respondents who had likely GAD were about three times more likely to present with a drug related problem compared to respondents who did not have a likely GAD when controlling for a mental health diagnosis and use of psychotropic medication before the wildfire.

**Table 5:** Predictors of substance use.

**Predictor**	**B**	**SE**	**Wald**	**df**	***P*-value**	**Odds ratio**	**95% CI for odds ratio**	
							**Lower**	**Upper**
**Respondent had no history of mental health diagnosis before the wildfire**								
No Yes	0.87	0.44	3.94	1	0.05	2.39	1.01	5.65
**Respondents were on psychotropic medication before the wildfire**								
No Yes	0.20	0.48	0.18	1	0.67	1.22	0.48	3.13
**Respondents had likely GAD after the wildfire**								
No Yes	1.06	0.34	9.63	1	0.002	2.88	1.48	5.62
**Constant**	−2.79	0.22	158.85	1	0.00	0.06		

Separate logistic regressions models however showed that likely GAD did not make unique statistical contributions to either hazardous drinking/alcohol dependence or moderate to high nicotine dependence when controlling for the presence of a mental health diagnosis and use of psychotropic medication before the wildfire.

## Discussion

This paper examined the prevalence and risk factors for GAD symptoms following the costliest natural disaster in Canadian history (Fort McMurray wildfire). Overall, the prevalence of generalized anxiety symptoms was high, with approximately one fifth of the sample reporting elevated symptomatology on self-report. This is approximately eight times higher than the rates of GAD in general Canadian population which was 2.5% in 2012 (31). Although the specific prevalence rate for GAD in our study population is unknown, it would be reasonable to assume it would not be much different from that of the general Canadian population. Furthermore, only about 14% of our study population self-reported a life time history of an anxiety disorder and only 9.6% were on an antidepressant. The prevalence rates for GAD symptomatology noted in our study are therefore significant, considering that the participants in the study were generally relatively high-functioning, with a minority endorsing pre-existing mental health conditions and the majority receiving support, with few relocations and direct property loss reported.

Clear risk factors emerged for elevated GAD symptomatology. Persons with elevated symptoms were more likely to be young, female, staying in rental or other accommodation, relocated to a different home, be exposed to media coverage relating to the wildfire, have pre-existing mental health conditions, and be receiving inadequate support.

Of the variables examined, the most salient predictors of elevated GAD symptomatology including witnessing of homes burning, relocation after the wildfire, a pre-existing anxiety disorder, level of perceived support from the government or family/friends, and whether post-crisis counseling was received. The presence of these variables was associated with an increased risk of GAD symptomatology, ranging from a two-fold (witness of homes burning) to a nearly seven-fold (pre-existing anxiety disorder) increased likelihood of elevated GAD symptoms. Increased social support has been reported in previous studies to protect against the mental health effects of post-natural disasters. In a longitudinal evaluation in a rural community sample in northern China after an earthquake, the group that received more support showed a general improvement in post-disaster well-being from 3 to 9 months(21). Similarly, another study reported that lower social support was associated with higher post-disaster psychological distress (32). Furthermore, the literature suggest that communal coping is protective against the mental health effects of the trauma associated with wildfires (15). The findings reported in these studies are consistent with our study results which suggest that increased support from Government, family and friends for victims could be protective against GAD after wildfires.

Our study data are also consistent with studies of mental health following other disasters, which have reported grossly increased prevalence rates as well as predictors of symptomatology that relate to pre-existing mental health conditions and exposure-related variables (32–35).

Our study suggested that elevated GAD symptoms were associated with increased or problematic substance use.

This paper extends the literature by demonstrating associations between GAD symptoms and these variables. GAD symptoms are often not the subject of examination post-disaster, yet they are highly prevalent and associated with relatively more persistence over time and far-reaching effects than other conditions.

The results of this paper suggest that post-disaster, it is important to screen residents for not only post-traumatic stress disorder and depression, but also (GAD). Results also suggest that healthcare systems, public health decision-makers, and governments should work to implement supportive measures post-disaster to mitigate mental health symptomatology.

Limitations are often unavoidable when working within the constraints of post-disaster conditions. There are therefore limitations of the study that merit mention. First, random sampling methods were used, thus the method of data collection was not systematic at a population level. Second, the sample of respondents was not fully representative of the community (e.g., over 66% of our respondents were female even though they comprise < 45% of the Fort McMurray population and we were unable to collect data from all categories of Fort McMurray inhabitants such as the workers in remote camps). Third, likely GAD was identified via screening measure self-report as opposed to a formal diagnostic interview. It is therefore possible that some respondents had anxiety associated with other mental health effects of wild fires including PTSD and Major Depressive Disorder rather than a GAD, Fourth, although we aimed for a sample of 1050 participants we were able to collect data from 488. This increased the margin of error of estimates (i.e., ±4.5% at 95% confidence intervals rather than the estimated ±3% margin of error). Notwithstanding these limitations, our study being one of the few studies to examine the mental health effects of the costliest natural disaster in Canadian history adds to the literature by documenting potential predictive factors for GAD symptomatology after wildfires. Knowledge of these factors would be helpful for policy makers when formulating social and clinical programs to mitigate the mental health effects of natural disasters.

## Conclusions

The results of the study suggest prevalence of (GAD) symptoms are grossly elevated following a natural disaster. There are risk factors that increase risk of elevated symptomatology. Specifically, a pre-existing anxiety disorder, witnessing the burning of homes during a wildfire, exposure to wildfire media coverage, relocation, and a perceived lack of governmental support or support from family/friends provide unique contributions to the prediction of elevated anxiety disorders following a wildfire. Substance use is also higher in persons with (GAD) symptoms. Policy implications include screening for generalized anxiety following disasters as well as provision of support to help mitigate the prevalence and impacts of post-disaster anxiety.

## Datasets are available on request

The raw data supporting the conclusions of this manuscript will be made available by the authors, without undue reservation, to any qualified researcher.

## Author contributions

VA conceived and designed the study, supervised data collection, analyzed the data and jointly drafted the initial manuscript with MH. MH contributed to the study design and analysis and jointly drafted the initial manuscript with VA. MJ participated in analyzing the data, reviewing and editing the initial draft of the manuscript. JO, ED, BN, IA, SM, and SC participated in data collection, reviewing and editing the initial draft of the manuscript. MB, PC, AG, and X-ML contributed to data interpretation and editing the initial draft of the manuscript. All authors approved of the final draft of the manuscript before submission.

### Conflict of interest statement

The authors declare that the research was conducted in the absence of any commercial or financial relationships that could be construed as a potential conflict of interest.

## Footnotes

^1^Fort McMurray wildfire: $3.6B in damage, says Insurance Bureau of Canada | Globalnews.ca., (n.d.). Available online at: https://globalnews.ca/news/2808805/fort-mcmurray-wildfire-cost-of-damage-from-alberta-disaster-to-be-released/ (Accessed March 5, 2018).
